# Lessons Learned in Research to Address Wicked Problems Facing the Care of Older Adults Across the Care Continuum

**DOI:** 10.1093/geroni/igaa057.2451

**Published:** 2020-12-16

**Authors:** Debra Dobbs, Sheryl Zimmerman

## Abstract

A wicked problem, by definition, has innumerable causes, is tough to describe, and doesn’t necessarily have a right answer. This describes today’s health care for older adults across the long-term care continuum. Our interdisciplinary group has almost as many years (n=70) of experience as GSA conducting research in community-based groups across the continuum of care from skilled nursing facilities, assisted living communities, adult day care, to independent living. In this symposium we will discuss lessons learned in recruitment, intervention delivery, and unexpected outcomes. Peterson will discuss lessons learned in using large data sets to derive actionable information on staff license mix and SNF complaints. Dobbs will discuss the utility of using hospice nurses to train ALC nurses in delivering palliative care. Lee will discuss lessons in engaging direct care workers in their need for sleep. Meng will discuss learning to embrace an unexpected finding that friendships developed in a dementia family caregiver music and mindfulness intervention were as meaningful as positive health outcomes. Buck will discuss lessons learned in recruiting a hidden group – informal caregivers with complicated grief. Finally, Zimmerman, as expert long-term services and support discussant, will pull the pieces together across the studies to facilitate discussion. Enrich your future research related to addressing wicked problems in health care for older adults by learning from our experiences.

